# Primary Vaginal Alveolar Rhabdomyosarcoma in an Adult Woman: A Case Report

**DOI:** 10.7759/cureus.108796

**Published:** 2026-05-13

**Authors:** Cijo Sanal, Isha Jaiswal, Lahunshisha Kharbuli, Pratikshya Biswal, Atreyee Chakrabarty

**Affiliations:** 1 Department of Radiotherapy, Institute of Medical Sciences, Banaras Hindu University, Varanasi, IND; 2 Department of Pathology, Institute of Medical Sciences, Banaras Hindu University, Varanasi, IND

**Keywords:** adult malignancy, alveolar rhabdomyosarcoma, case report, gynaecologic oncology, multimodal treatment, rare gynecologic malignancy, rhabdomyosarcoma, soft tissue sarcoma, vaginal neoplasms, vaginal tumors

## Abstract

Rhabdomyosarcoma (RMS) is the most common soft tissue sarcoma in children, but its occurrence in adults is exceedingly rare. Among its subtypes, embryonal RMS predominates in the female genital tract, whereas alveolar rhabdomyosarcoma (ARMS) is exceptionally rare at this site, with only isolated cases reported in the literature. We describe the case of a woman in her mid-40s who presented with abnormal vaginal bleeding and a friable polypoidal mass arising from the anterior vaginal wall. Magnetic resonance imaging demonstrated a well-defined lesion measuring approximately 28 × 23 × 13 mm, confined to the vaginal wall, without invasion of adjacent structures or evidence of nodal or distant metastasis. The patient underwent wide local excision of the lesion. Histopathological examination revealed a malignant small round cell tumor with an alveolar growth pattern, and immunohistochemistry confirmed the diagnosis of ARMS, showing strong nuclear positivity for myogenin and cytoplasmic positivity for desmin, with negative epithelial, lymphoid, and neural markers. Following multidisciplinary tumor board discussion, the patient was planned for adjuvant systemic chemotherapy with ifosfamide and adriamycin, followed by external beam radiotherapy (50 Gy in 25 fractions) and intracavitary brachytherapy boost (6 Gy in three fractions). Adult vaginal ARMS is associated with an aggressive clinical course and poor prognosis despite multimodal therapy. This case highlights the importance of considering RMS in the differential diagnosis of vaginal masses in adults and underscores the need for early diagnosis and multidisciplinary management in improving outcomes.

## Introduction

Rhabdomyosarcoma (RMS) is a malignant soft tissue sarcoma arising from primitive mesenchymal cells committed to skeletal muscle differentiation. It represents the most common soft tissue sarcoma in children and adolescents; however, its occurrence in adults is rare and is generally associated with poorer clinical outcomes compared with pediatric patients [1]. Although RMS can arise at various anatomical sites, involvement of the female genital tract is uncommon, where the embryonal subtype, particularly sarcoma botryoides, is most frequently encountered in pediatric patients. Alveolar rhabdomyosarcoma (ARMS) involving the vagina is exceedingly rare in adults and has been reported only in isolated cases [2]. ARMS is characterized by a more aggressive clinical course, a higher propensity for early metastasis, and an overall unfavorable prognosis compared with other subtypes [1]. Because of its rarity in adults, optimal diagnostic and therapeutic strategies are not well defined and are largely extrapolated from pediatric cooperative group protocols [1]. Reporting such rare presentations is important to expand the existing literature, emphasize the diagnostic role of histopathology and immunohistochemistry, and contribute to improved management strategies for adult patients presenting with vaginal neoplasms.

This work was previously presented as a poster at the Annual Conference of the Uttar Pradesh Chapter of the Association of Radiation Oncologists of India (AROI-UP Chapter) on November 8-9, 2025.

## Case presentation

A 45-year-old woman with no significant comorbidities presented in April 2025 to our tertiary care center with abnormal vaginal bleeding of six-month duration. The bleeding was initially intermittent but progressively became persistent and was associated with abdominal discomfort and dull aching lower back pain. She also reported foul-smelling vaginal discharge at symptom onset without any urinary or bowel complaints.

Her past medical history was notable for a hysterectomy performed two years earlier for abnormal uterine bleeding. There was no history of prior malignancy or significant family history of cancer.

On general examination, she was moderately built and nourished, with a body mass index of 22.5 kg/m² and a Karnofsky Performance Status [3] of 90%. No palpable lymphadenopathy was detected. Local examination revealed a friable polypoidal growth measuring approximately 4 × 3 cm, arising from the anterior vaginal wall about 1 cm below the urethral meatus. The lesion had a broad stalk and bled on contact. Per rectal examination showed no rectal wall induration, and abdominal examination was unremarkable.

**Figure 1:**
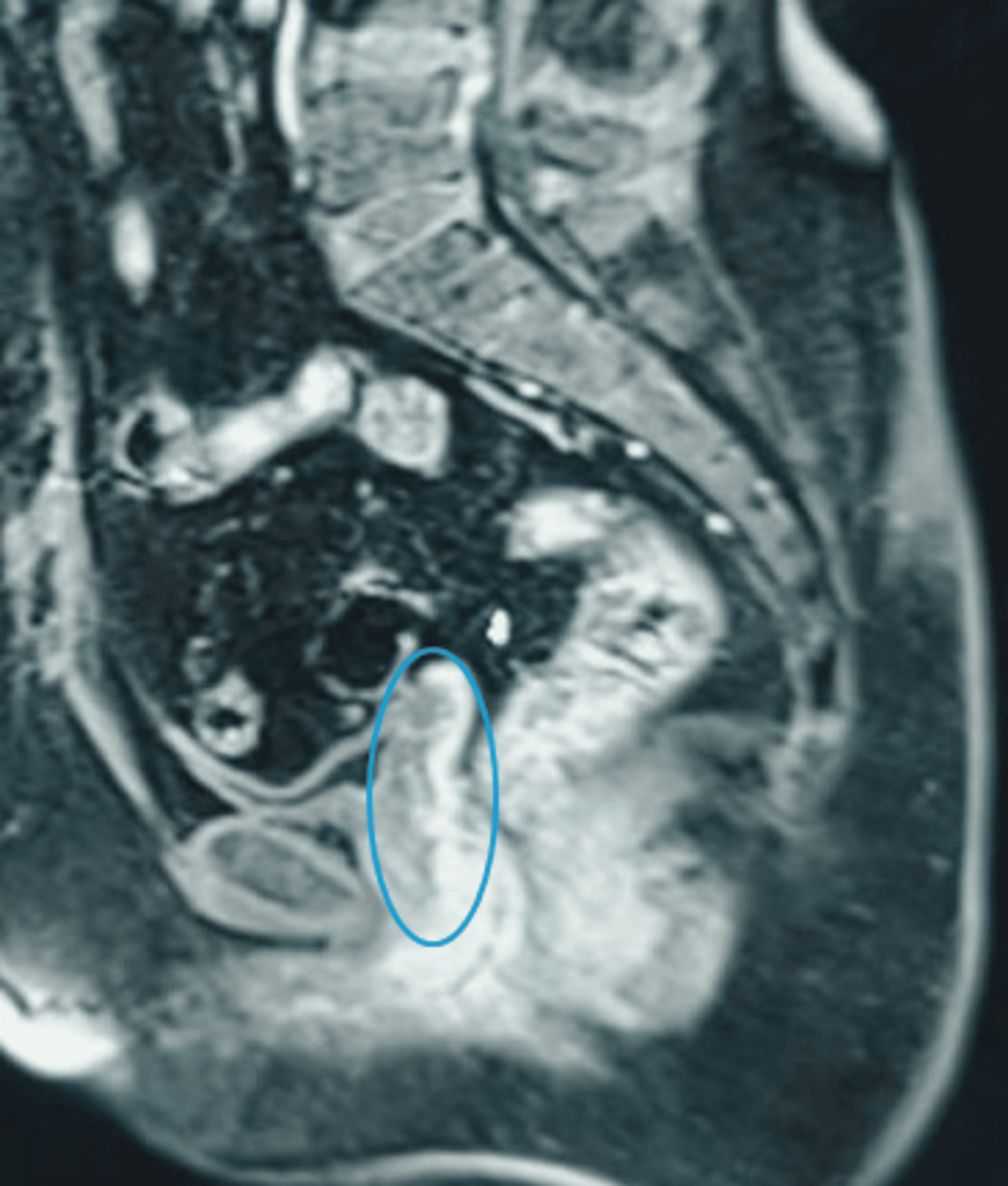
Sagittal T2-weighted MRI of the pelvis showing anterior vaginal wall mass Sagittal T2-weighted MRI of the pelvis showing a well-defined polypoidal mass arising from the anterior vaginal wall (encircled) without adjacent organ invasion.

Baseline hematological and biochemical investigations were within normal limits. Magnetic resonance imaging (MRI) of the pelvis demonstrated a well-defined polypoidal mass measuring 28 × 23 × 13 mm arising from the anterior vaginal wall with a broad-based attachment (Figure 1).

The lesion appeared hypointense on T1-weighted images and hyperintense on T2-weighted images, with moderate post-contrast enhancement. There was no evidence of invasion into the bladder, rectum, or parametrial tissues, and no pelvic or para-aortic lymphadenopathy was identified. Contrast-enhanced computed tomography (CECT) of the chest showed no pulmonary or mediastinal metastases. A comprehensive metastatic workup did not reveal any primary lesion elsewhere, supporting the diagnosis of a primary vaginal malignancy.

**Figure 2:**
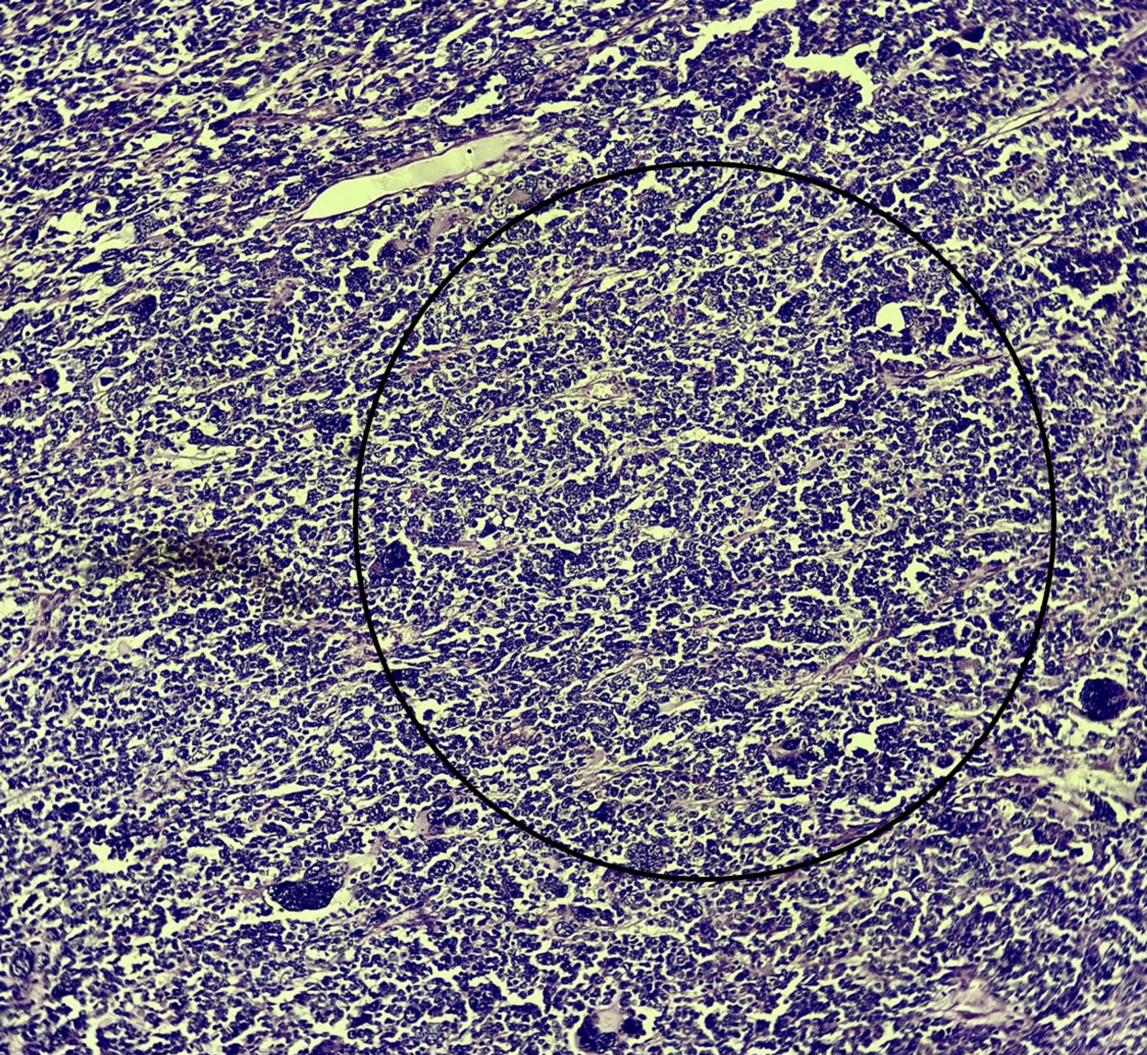
Low-power photomicrograph showing alveolar architecture of the tumor Low-power hematoxylin and eosin (H&E) stained section (10×) demonstrating a highly cellular malignant neoplasm arranged in an alveolar growth pattern (encircled), characterized by nests of tumor cells separated by delicate fibrovascular septae.

**Figure 3:**
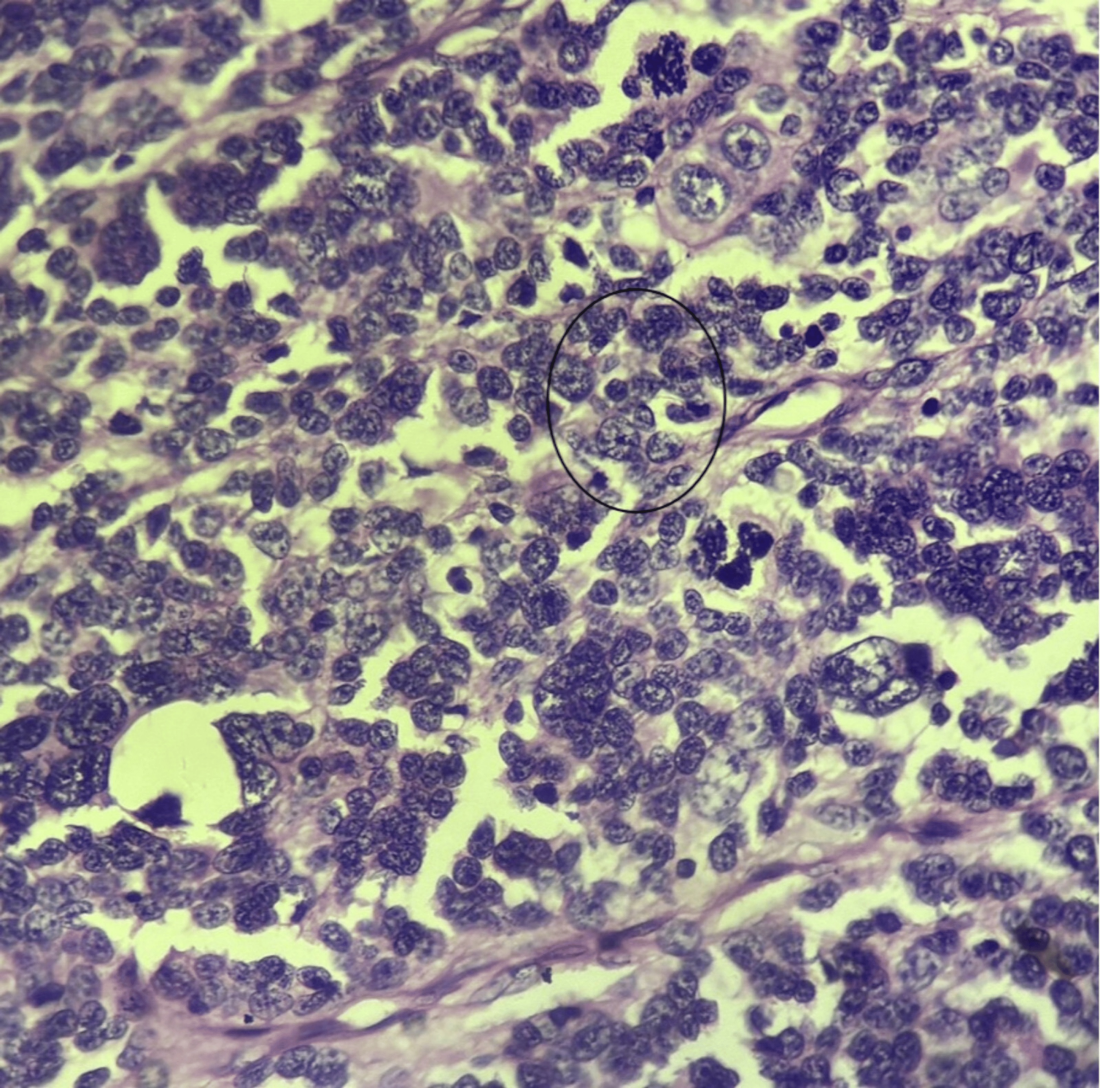
High-power photomicrograph demonstrating cytological features of alveolar rhabdomyosarcoma High-power hematoxylin and eosin (H&E) staining photomicrograph (40×) demonstrating sheets of malignant small round cells with hyperchromatic nuclei, prominent nucleoli, and moderate cytoplasm, consistent with alveolar rhabdomyosarcoma.

The patient underwent wide local excision under general anesthesia via a vaginal approach, achieving clear surgical margins. No intraoperative complications were noted. Histopathological examination revealed a high-grade malignant neoplasm composed of small round cells arranged in an alveolar growth pattern. The tumor cells demonstrated moderate pleomorphism, round to oval hyperchromatic nuclei, vesicular chromatin, conspicuous to prominent nucleoli, and scant to moderate cytoplasm. Multiple congested vascular channels were present in the intervening stroma (Figures 2, 3).

Immunohistochemistry showed diffuse strong nuclear positivity for myogenin and cytoplasmic positivity for desmin, confirming the diagnosis of alveolar rhabdomyosarcoma. The tumor cells were negative for pan-cytokeratin (PanCK), leukocyte common antigen (LCA), and soluble in 100% ammonium sulfate (S100), excluding epithelial, lymphoid, and neural neoplasms. In view of these findings, the disease was classified as a localized vaginal soft tissue sarcoma confined to the vaginal wall, with no evidence of regional nodal or distant metastasis.

The case was discussed in a multidisciplinary tumor board meeting, and adjuvant multimodal therapy was planned. The patient was initiated on six cycles of systemic chemotherapy with ifosfamide and adriamycin administered intravenously every three weeks, followed by planned external beam radiotherapy to a dose of 50 Gy in 25 fractions (2 Gy per fraction) to the primary tumor bed with appropriate margins. This will be followed by a boost of 6 Gy in three fractions using intracavitary brachytherapy, ensuring adequate coverage of the tumor bed while sparing surrounding organs at risk. The patient has tolerated treatment well without significant therapy-related complications to date.

The patient is planned for regular follow-up with clinical and radiological evaluation every three to six months.

## Discussion

RMS is an uncommon malignancy in adults, accounting for a small proportion of adult soft-tissue sarcomas [1]. Involvement of the female genital tract is rare, and when present, embryonal histology, particularly the botryoid subtype, is more frequently encountered in children. In contrast, ARMS arising in the vagina of adults is exceptionally uncommon, with only isolated cases described in the literature [2,4]. Adult cases reported in the literature consistently emphasize the rarity of this entity [2,4], while population-based analyses indicate more aggressive clinical behavior and poorer outcomes in adults compared with pediatric patients [1]. Each additional report contributes to a better understanding of its clinical behavior and management.

Histologically, ARMS is characterized by nests of small round cells separated by fibrous septa, with central discohesion producing the classic alveolar pattern. Immunohistochemically, strong nuclear myogenin expression and cytoplasmic desmin positivity support the diagnosis [5]. Molecular studies frequently demonstrate PAX3/7-FOXO1 fusion transcripts, which are associated with aggressive tumor biology and inferior clinical outcomes [5]. However, molecular confirmation could not be performed in the present case due to resource limitations, which is acknowledged as a limitation of this report.

Unlike pediatric RMS, there are no management guidelines specifically tailored to adults. Treatment strategies are largely extrapolated from pediatric cooperative group protocols and typically include multimodal therapy consisting of surgery, systemic chemotherapy, most commonly vincristine, actinomycin-D, and cyclophosphamide (VAC), and radiotherapy when indicated [1]. However, while VAC-based regimens are standard in pediatric RMS, there is no consensus regarding optimal chemotherapy in adults. Adult patients are often treated using soft tissue sarcoma protocols, and regimens such as ifosfamide and doxorubicin are commonly employed, considering differences in tumor biology and treatment tolerance. The choice of chemotherapy in the present case was therefore based on multidisciplinary tumor board recommendation. Furthermore, adults often present with more advanced disease, tolerate chemotherapy less well, and demonstrate inferior survival compared with children, with reported five-year overall survival rates ranging from 20% to 30% in adults, whereas survival in pediatric patients exceeds 70% in large population-based analyses [1].

Published adult cases consistently highlight the aggressive clinical course of ARMS. Vaginal and vulvar cases have demonstrated early recurrence and rapid distant metastasis despite multimodal treatment, including surgery and VAC-based chemotherapy [2,4,6]. Extragenital adult presentations similarly report high rates of metastatic spread involving the lungs, bones, peritoneum, and retroperitoneum, often leading to limited survival even with aggressive therapy [7-10]. Although rare favorable outcomes have been described following complete surgical excision combined with systemic chemotherapy and radiotherapy [11,12], these remain exceptions. Overall, the prognosis in adults remains generally poor, as consistently reported across published case series and population-based analyses [1-4,6].

In contrast to many previously reported cases, our patient presented with localized disease without nodal or distant metastasis, enabling complete surgical excision followed by planned adjuvant multimodal therapy. Early-stage detection may offer a potential survival advantage in selected adult patients [1]. Increased clinical awareness of this rare entity, particularly in adult women presenting with abnormal vaginal bleeding or polypoidal vaginal lesions, is essential. Prompt evaluation with imaging and early biopsy of suspicious lesions can facilitate timely diagnosis, and maintaining a broad differential diagnosis for vaginal masses may help avoid delays in recognition. Nevertheless, given the well-documented metastatic propensity of adult ARMS, close long-term surveillance is essential to detect recurrence or distant spread [1,2].

## Conclusions

Primary ARMS of the vagina in adults is an exceptionally rare and aggressive malignancy. Accurate diagnosis requires integration of clinical, radiological, histopathological, and immunohistochemical findings. Multimodal management, including surgery, systemic chemotherapy, and radiotherapy, remains the cornerstone of treatment, although outcomes in adults are generally inferior to those in pediatric patients. In the present case, early-stage localized disease allowed complete surgical excision followed by planned adjuvant multimodal therapy, which may offer a potential advantage in selected patients. Early recognition and close long-term surveillance are essential given the high propensity for distant metastasis.
